# Spirituality Concept by Health Professionals in Iran: A Qualitative Study

**DOI:** 10.1155/2016/8913870

**Published:** 2016-07-17

**Authors:** Nadereh Memaryan, Maryam Rassouli, Maryam Mehrabi

**Affiliations:** ^1^Academy of Medical Sciences of Islamic Republic of Iran, School of Behavioral Sciences and Mental Health, Iran University of Medical Sciences, Tehran 1443813444, Iran; ^2^Nursing & Midwifery School, Shahid Beheshti University of Medical Sciences, Tehran 1467664961, Iran; ^3^Department of Mental, Social Health and Drug Abuse, Ministry of Health, Tehran 1919973361, Iran

## Abstract

*Background*. For years, researchers have sought to provide a clear definition of spirituality and its features and consequences, but the definitions provided of this concept still lack transparency. The present qualitative research was conducted to clarify this concept within the religious-cultural context of Iran.* Materials and Methods*. The present conventional qualitative content analysis was conducted with an inductive approach. Data were collected through semistructured interviews with 17 spiritual health experts and activists selected through purposive sampling.* Results*. Three themes emerged from the analysis of the data, including (1) the structure of spirituality, (2) defects in the conceptualization of spirituality, and (3) spirituality in practice, which are explained in this paper with their relevant subthemes and codes. The definition which this study proposes for this concept is that “spirituality is the sublime aspect of human existence bestowed on all humans in order for them to traverse the path of transcendence that is closeness to God (Allah).”* Conclusion*. The definition provided by this study is similar to the previous definitions of this concept in its main part (transcendence) and in incorporating a God-centered view of spirituality within the context of an Islamic society. This definition has implications for health services' education, research, and practice in similar societies.

## 1. Introduction

As a valuable divine blessing, health is defined by both a physical and a spiritual dimension in Islam, with the latter being even more important [1]. Studies have shown the significant effect of the spiritual dimension on the physical and mental dimensions of health [2]. Nevertheless, medical literature does not yet have a classic definition for spirituality [3], as this concept is still discussed with a haphazard mixture of definitions [4] that have undergone a series of changes within decades [5].

The increasing attention to the concept of spirituality, particularly with respect to health, is the result of the extensive range of data produced by empirical studies indicating the effect of spirituality on health [6]. Nevertheless, the concept of spirituality cannot be used in health services unless its meaning is further clarified [7]. Researchers working on the subject also need to reach a specific definition for the concept [8]. Some studies consider spirituality a measurable concept, while others believe it cannot be measured [9].

Differences in the definitions provided in literature of spirituality may also be attributed to the cultural differences in place [10]. This concept may also be affected by the ethnicity, living environment [11], cultural background, religious beliefs, and experiences of the researcher who analyzes and interprets the data pertaining to the conceptualization of spirituality [6].

The majority of studies that deal with spirituality as a main concept have failed to provide a clear and concise definition of the concept and have instead resorted to giving a mere description of it [6]. For years, researchers have sought to provide a clear definition of this concept and its features and consequences [12, 13]. Examining the relationship between this concept and religion is a major challenge for researchers [14, 15], especially since the definitions provided of this concept still lack transparency [3] and since it is very often mistaken for religion and used interchangeably or in combination with it [11].

According to a review of studies conducted between 1999 and 2013 on spirituality and religion in health [16], Iran ranks high in terms of producing scientific literature on the subject. Nevertheless, there are still only a limited number of studies that have been dedicated to extracting the meaning of spirituality [17], and studies conducted on spirituality and health in Iran have failed to provide a localized definition for the concept [18–21]. As a result, despite all the research and the need felt by Iranian health professionals for the use of this concept in health services, there are no protocols and guidelines for the use of spirituality in practice [22]. Although some hospitals in Iran have Islamic clergies to provide related care, developing guidelines for integrating spirituality in mainstream healthcare setting is a necessary need. Also in the medical sciences education, spirituality-related education is not currently observed in medical sciences' curriculum in Iran and the lack of consensus on a precise definition for spirituality in Islamic context is one of the main challenges to the decisions to include spirituality in medical education curricula [23] as well as find practical model and linked guidelines [24].

The method of qualitative content analysis was used to clarify this concept within the religious-cultural context of Iran, as it is often used to present a concept from a conceptual framework. The inductive approach to this method helps present fragmented phenomena [25]. The present study uses inductive qualitative content analysis to present the structure of the concept of spirituality, the defects for providing a comprehensive definition of the concept, and the practical approaches to its implementation in health services based on the qualitative data extracted from local spiritual health experts and activists. The authors believe that presenting this conceptual framework can help expand the applied research on the subject, including the concept and its consequences assessment in practice, and also can present a definition for spirituality in Islamic context that leads to growth and development of spiritual services and education in Iran and the other countries with a similar context.

## 2. Materials and Methods

The present study is a conventional qualitative content analysis conducted with an inductive approach to provide a definition for the concept of spirituality from a conceptual framework. The inductive approach to content analysis facilitates the description of concepts which the existing theories and literature fail to agree on their definition [25, 26].

Given that interviews were used to collect the data, this section of the paper examines the COREQ-32-item checklist for each subtitle. This checklist is designed for qualitative studies that use interviews or focus groups [27].

### 2.1. Research Team

The first author of this paper conducted most of the interviews. She is a holder of an M.D. degree and a Community Medicine Specialist and was a Member of the Academy of Medical Sciences of Iran at the time of carrying out this project; she, moreover, has experiences in conducting qualitative research (items 1 to 5 of the COREQ-32-item checklist). All the participants were contacted over the phone to set up a time for conducting the interviews and were also briefed on the study objectives at the beginning of the interview sessions (items 6 to 8 of the COREQ-32-item checklist).

### 2.2. Participants

The present content analysis yielded rich data on the subject by using purposive sampling to select participants from among spiritual health activists or published experts of the field. Efforts were made to ensure sufficient sample variation with respect to the education and occupation and position in spiritual health services through examining publications and participants in relevant meetings, conferences, and seminars. Arrangements were made to conduct 17 interviews in either the interviewee's or the project executive's workplace under quiet and undisturbed conditions. Face-to-face interviews were conducted with no other person present in the room. There were no refused or dropped-out cases. Of the total of 17 participants, 12 (70%) were male and 5 (30%) were female. All of them were Muslim with an average age of 46.8 (±13.5) years.  Table 1 presents participants' information, including gender, degree of education, and occupation and position in spiritual health services (items 9–16 of the COREQ-32-item checklist).

### 2.3. Data Collection

Data were collected through semistructured interviews with questions including the following. “What does spirituality mean to you?” “How do you define spirituality?” “What is your understanding of this concept?” Field notes were taken and the interviews were audio-recorded with participants' consent and were then transcribed and entered into a computer at the first opportunity. The interviews were conducted only once with each participant, lasted for 30–95 minutes, and continued until data saturation was reached and no new codes were emerging any longer (items 17–22 of the COREQ-32-item checklist).

### 2.4. Data Analysis

The text of each transcribed interview was taken as the unit of analysis and was then analyzed in five stages according to the model proposed by Granheim and Lundman [28]: (1) reading the text several times to grasp general concept, (2) dividing the text into meaning units, (3) condensing and shortening the meaning units by including the most important core, (4) designing the codes that were abstracted and labeled of condensed meaning unit, and (5) considering the similarities and differences between codes for developing the subthemes and themes. Since the qualitative analysis requires a cyclic process [29], we would repeatedly return to complete the stages.  Table 2 presents some examples of some stages of the analysis.

No participant checking was performed, but efforts were made to extract clear themes and subthemes that were inclusive of all the relevant codes which were coded by one data coder. Data management was done manually. Moreover, direct quotations were frequently included in the Results section of the paper (items 24–27 and 29–32 of the COREQ-32-item checklist).

### 2.5. Data Rigor and Trustworthiness

In addition to meeting 30 out of the 32 criteria of the COREQ-32-item checklist, the rigor and trustworthiness of the data was ensured through immersion in the subject, peer checking, and data source triangulation using experts from different fields to collect the data [23]. For conducting the peer review, each interview was first coded by the first author and then reviewed by the second author and some codes were modified if necessary. Moreover, the charts extracted by the first author were checked by the second author in the middle and late stages of the analysis. Data source triangulation was carried out, since our participants were selected from different areas of education.

### 2.6. Ethical Considerations

The data collected in the present study are part of the data obtained for a research project approved by the Academy of Medical Sciences of Iran under Contract number F/A/P/1/5690. The study participants submitted their informed consent forms after they were briefed on the study objectives and were ensured of the confidentiality of their information (achieved through the anonymous publication of the results) and their right to withdraw from the study at any stage. Ethical principles and participants' rights were observed in carrying out the interviews.

## 3. Results

Three themes emerged from the analysis of the data, including the structure of spirituality (Table 3), defects in the conceptualization of spirituality (Table 4), and spirituality in practice (Table 5), all of which are further explained in this paper with their relevant subthemes and codes. A definition of spirituality extracted by the authors based on the data obtained is then presented.

1. * The Structure of Spirituality*. The structure of spirituality was the first theme extracted from the data and includes the nature of spirituality, components of spirituality, and characteristics of spirituality as its subthemes.
  (1.1) * The Nature of Spirituality*. This subtheme contains all that is naturally inferred from spirituality. It is the dimension of humans' existence that is most influential in their life, involves a search for meaning and the sense of belonging to a superior essence beyond the self, that is, God, entails humans' understanding of the Creation and the meaning of life, and is considered a great source of strength for them. One participant expressed her understanding of spirituality as follows: “I divide life into two inseparable and totally related parts; one is the protection of the body and material resources, including the human and the environment that is bestowed on him, and the other is that which is called* spiritual life* in the Quran. Man is beyond mere flesh and the health associated with that part of him is much more helpful” (P2). Another participant revealed the following: “The first thing that comes to my mind about the issue of spirituality is to ask What is human? What is he made of? What is the world [made of]? What am I made of? Where am I supposed to be headed? How am I connected to the world of existence? Why am I here?” (P4).
  (1.2) * Components of Spirituality*. The components or the constituent parts of this concept include connection with God, the self, the others, and even the nature. One participant commented as follows: “Self-knowledge, theology and connection with the others are in fact the core of spirituality. And the nature is another issue. I believe that the nature brings man closer to God, and that is how he can get closer to God” (P9).
  (1.3) * Characteristics of Spirituality*. This subtheme implies the characteristics that somehow distinguish the concept of spirituality from other routine concepts in health. These characteristics include being abstract, personal, and variable, not being specific to any one area of science, and growing in accordance with the individual's own understanding and perception. To quote some participants on the subject, they reported the following. “As spirituality, this entity is highly personal and unique. This concept is also very personal, so much that even the prophet should utter ‘Glory be to God'. No matter what concept you take, Glory still be to God. Even within twenty years, this concept will still continue to change” (P1). “Spiritual matters are a bit abstract and presumably inaccessible. You have to work at them for a long time, I think, and not sporadically” (P13).
2. * Defects in the Conceptualization of Spirituality*. The next theme that emerged from the data was defect, consisting of the three subthemes of challenges related to the concept, necessity for spirituality conceptualization, and measurement of spirituality.
  (2.1) * Challenges Related to the Concept*. Within the cultural context of a country such as Iran, God-orientation is inseparable from the concept of spirituality, comprising a major challenge due to the different beliefs held by the people. There are other challenges too, including the different perspectives on spirituality as either a means of living a good life or the main purpose in life, the concept not having a distinctive meaning and instead bordering on similar concepts, especially concepts in ethical practice, the distortion of certain subset terms, and, finally, its confusion with certain psychology terminology (such as using the spirit and the psyche in combination). A Psychiatrist Faculty Member commented as follows: “Spirituality proposes a dimension for the human existence that necessitates a sort of belonging to a higher being than the self. The dispute is over belonging to something superior and higher-God in our culture; but this perception does not necessarily hold true for all people. It cannot be said that spirituality necessarily involves God in all religions” (P1). Another participant who is a University President said the following: “In the secular view, physical health comes first and psychological health comes second. Spiritual health is therefore desired for physical health, and if they have to sacrifice one over the other, it will be spiritual health that is sacrificed. For us, though, spiritual health is the core. Fasting is not harmful for the body, but even if some day they discover that it is harmful, we won't give up fasting, because it develops spiritual health” (P7).
  (2.2) * Necessity for Spirituality Conceptualization*. Knowing the human conditions, having transparency in providing definitions, and being able to differentiate the concept from concepts in the fields of psychology and ethics are essential. Participants made different comments: “Since spiritual health concerns humans, we should first understand and define the human life. People who have examined the human life have no doubts that humans have both a physical and a metaphysical dimension. In other words, everyone agrees that humans have a corporeal and an ethereal dimension. The ethereal dimension is identical to the spiritual dimension” (P3) and the other participant said the following: “What exactly are spiritual behaviors? What are their social consequences? We should differentiate between ethics, the psyche and spirituality. We should make clear distinctions between them and then examine what makes a behavior spiritual; for example, praying” (P13).
  (2.3) * Measurement of Spirituality*. Participants noted the immeasurability of the concept of spirituality and therefore its consequences in their discussion of this subtheme. “Non-corporeal beings are measured by their effects and outcomes. For instance, when we refer to someone as a great person, we are not thinking about their size, but about their distinguished attributes; for example, their being a scientist or an inventor” (P3).
3. * Spirituality in Practice*. This theme has three subthemes, including consequences of spirituality, manifestations, and levels of spirituality, which refer to spirituality in a practical way.
  (3.1) * Consequences of Spirituality*. The emergence of spirituality in human life leads to consequences such as satisfaction with life, surrendering to the will of God, meaningful experiences of pain and suffering in life, and lasting peace of mind. One nurse stated the following: “We had heart and lung transplant patients at the ward and my duty was to take care of them. There was, for instance, a lung transplant patient who had sold all that he had to pay for the operation, his family was under a lot of pressure, and they knew that the transplant would be rejected within 6 months; yet, he and his family were still happy and smiling; when I asked his family why that was the case, I realized how more spiritual they were in character” (P5). Another participant who was a Faculty Member remarked as follows: “Well, we consider reaching spiritual health a priority for everyone, because not only does it ensure health in this world, but also in the world beyond” (P17).
  (3.2) * Manifestations of Spirituality*. The codes obtained in this subtheme are manifestations of spirituality, such as patience, faith, sacrifice, hopefulness, respect, and ethics. One Medical Student said the following: “In our field, for instance, we consider humanism, ethics and altruism parts of spirituality. For example, helping a patient when you are exhausted is part of being a spiritual person. This is my definition of spirituality. A lot of spirituality fits into our religion. I believe that professional ethics are also part of spirituality” (P10). An experienced Psychiatrist noted the following: “When I visit patients, I try to look for a manifestation of spirituality in them; I find healing, faith, praying, fasting, worship, abstinence from forbidden foods and alcohol, observing religious obligations, rituals and the tradition all constitute spirituality; yet, I should be careful as to which of them is considered pathological?” (P12).
  (3.3) * Levels of Spirituality*. Levels are the most important concept in this subtheme. People are born with healthy spirituality, and then comes religiosity, which determines how people traverse the levels of spirituality. An expert in religious studies said the following: “Physically, people may be born with illness. Spiritually? Never. This is key” (P14). Another published expert in religious studies noted the following: “Spiritual health is very much related to religiosity. In fact, we believe that spirituality, in the actual sense of the word, cannot be properly achieved unless through religion. I think there are several parts to spiritual health, and if we divide it into its constituent signs, we find that each and every sign of spirituality can be found in religion. And that is truly the case, but it does not mean he cannot have any part of spiritual health” (P11).

*Definition of Spirituality*. We summarize the findings result in the following definition: “Spirituality is the sublime aspect of human existence bestowed on all humans in order for them to traverse the path of transcendence that is closeness to God (Allah).” Accordingly, the concepts of spirituality and religion are thus inseparable, although not in the sense that they are one and the same, but in the sense that religion is necessary for spiritual improvement.

## 4. Discussion

The present study provides a framework for spirituality that contains its structure and practical aspects as well as the defects that persist in the exploration and application of the concept. In addition, a definition is provided of spirituality based on the inductive analysis of the data.

The wide varieties of definitions provided to date for spirituality are all common in some aspects, including their specificity for every individual and their encompassing of a greater scope compared to religion. Nearly all the definitions provided are common in the transcendental dimension they ascribe to spirituality, the consequent search for the meaning of life, and the connection formed with the self, the others, the nature, and/or the higher being [9]. The definition provided in this study incorporates these aspects too; however, there is a fundamental difference that distinguishes the Iranian perspective on the concept of spirituality. According to Puchalski et al.'s [30] report of the meetings held with experts from all over the US and Europe to reach a consensus regarding the definition of this concept, spirituality has components such as the self, the others, the nature, transcendence, and sacredness. Nevertheless, these reports make no note of God, despite probably constituting the main component of the concept in Iran, according to the results of this study.

One of the themes extracted in a similar study conducted by Vahedian-Azimi and Rahimi in Iran concerns “the differences in the meaning and concept of spirituality from the perspective of individuals” [17], which is also noted in the present study as one of the codes pertaining to the challenges subtheme. Another study extracted Iranian nurses' view on the concept of spirituality [31] and found it to be closely tied to the nurses' religious beliefs, which is mostly founded on the belief in God. Evidence suggests that the context in which a concept is examined and the researcher who analyzes the data affect the definition that emerges in the society of the concept in question [6, 10, 11].

The subthemes presented in this study for the first theme (i.e., the structure of spirituality), including the nature, components, and features, are consistent with the findings of many other studies conducted on this subject. In a review study, Lepherd reports that spirituality is the existential core of humans. Spirituality is a personal matter that differs from one person to the next and is possessed by people of all religions and faiths [11]. In a descriptive and exploratory study, Chaves and Gil proposed spirituality as a great source of support, which supports the results obtained in the present study with regard to the subthemes of the nature and features [13]. A concept analysis model proposed by Buck integrates the four connections with God, the self, the others, and the nature presented in this study as the components of spirituality and thereby presents the integrated model of spirituality [32]. Studies conducted by Lepherd and Wong and Yau also confirm these connections [11, 33]. As noted, most definitions of spirituality are concerned with these four connections [9].

Several studies have examined the distinction between this concept and religion, including the study by Joshanloo [34], who conducted a factor analysis of the concept in a sample population of Shiite Muslims on the grounds that nearly all the empirical studies examining the relationship between “spirituality” and “religion” have been conducted in western cultures. Joshanloo concluded that the model that links these two constructs and yet distinguishes between them is preferred. The study by Wong and Yau [33] has also confirmed the more extensive scope of the concept of spirituality compared to religion, which is consistent with the codes extracted for the second theme obtained in the present study.

The third theme extracted from the data obtained, which is more concerned with spiritual health, is the product of the formation of spirituality in humans and its consequences, manifestations, and levels. In his review study, Lepherd refers to peace of mind, satisfaction, relief from suffering, and the experience of love as consequences and forgiveness, worship, and rituals as behavioral manifestations of spirituality [11]. In their concept analysis, Delgado and Mahlungulu and Uys also offered consequences for spirituality, including peace of mind, successful adaptation, guarantee for health, hope, and the meaningfulness of life [35, 36], which is consistent with the results of the present study.

What was proposed in the present study based on the views expressed by spiritual health experts and activists in Iran also proposed different levels for spirituality that are realized only when actually manifested in the insight, tendencies, and behaviors of humans; these levels involve humans being born with spiritual health and then, depending on their degree of adherence to religion, traversing the path of transcendence, culminating in the closeness to God.

## 5. Conclusion

The present qualitative content analysis analyzed the views of experts and scholars to provide a definition for spirituality and thus presented its framework as consisting of the structure of spirituality, defects, and spirituality in practice. The study then examined the relationship between this concept and religion and also the points of similarity and distinction between this definition and the ones provided by other researchers.

The results of the present study can have a positive impact on health services' education, research, and practice in Iran and in similar societies, with respect to religious beliefs. Given the need for reaching a consensus on the definition of concepts which are supposed to enter clinical services and be welcomed by providers, including physicians, nurses, and other healthcare providers, the findings of this study can be used for the development of a common language in spirituality. Exploration of this concept that was missing link in spiritual service and education in our healthcare setting should be led to clinical guideline development and curriculum planning in this field.

The limitations of the study included the lack of access to the English translation of the Persian and Arabic resources consulted. Several articles have been already written on the subject of spirituality in Persian; however, they were not examined and referred to due to the unavailability of their text or abstract in English and due to not being cited in general databases. Another limitation of the study is the impossibility of the generalization of the results, which is considered an inherent limitation of qualitative research. Since all the study participants were Muslim, the results may not hold true for individuals from other religions.

Further studies are recommended to be conducted on the subject in order to explore this concept and extract its indicators within different religious-cultural contexts. The use of localized definitions within each unique context enriches practical research on the subject and facilitates its interpretation.

## Figures and Tables

**Table 1:** Participants' information.

Number	Gender	Education	Occupation/position
1	Male	Psychiatrist	Faculty Member/Published Author
2	Female	Clinical Subspecialist	Faculty Member/Activist and Enthusiast
3	Male	Pharmacologist	Faculty Member/Activist and Enthusiast
4	Male	General Practitioner	Physician/Activist and Enthusiast
5	Female	M.S. in Nursing	Instructor/Published Author
6	Female	Medical Education	Ph.D. Student/Published Author
7	Male	Epidemiologist	Faculty Member/Activist and Enthusiast
8	Male	Clinical Specialist	Faculty Member/Activist and Enthusiast
9	Female	Ph.D. in Nursing	Faculty Member/Published Author
10	Female	General Medicine	Student/Activist and Enthusiast
11	Male	Ph.D. in Religious Studies	Faculty Member/Published Author
12	Male	Psychiatrist	Faculty Member/Published Author
13	Male	Community Medicine Specialist	Faculty Member/Activist and Enthusiast
14	Male	Ph.D. in Religious Studies	Faculty Member/Published Author
15	Male	Medical Ethics Specialist	Faculty Member/Published Author
16	Male	Psychologist	Faculty Member/Published Author
17	Male	Clinical Subspecialist	Faculty Member/Published Author

**Table 2:** Examples of meaning units, condensed meaning units, and codes.

Meaning unit	Condensed meaning unit	Code
Since spiritual health refers to humans, then we must first know humans correctly and also define and describe humans properly	Correct knowledge of humans: the first step in understanding the concept	Knowledge of humans
We have not reached a consensus on the definition of this concept. We must reach a consensus on this concept	The need for a consensus on the definition of the concept	Consensus

**Table 3:** The codes and subthemes pertaining to the theme of the structure of spirituality.

Theme	Subtheme	Code
The structure of spirituality	The nature of spirituality	A dimension of existence
		Belonging to God
		A great source of strength
		Purpose of Creation
		Meaning of life
		Search for meaning
		The ultimate goal of humans
		The influential part of human life
		Belonging to an eternal life
		Natural human need

The structure of spirituality	Components of spirituality	Connection with God
		Connection with the self
		Connection with the others
		Connection with the nature

The structure of spirituality	Characteristics of spirituality	Dynamic and changing
		Personal and individual
		Subjective and abstract
		Hierarchical
		Relativity
		Nonspecificity to a field of science
		Nonobjectivity
		Growing

**Table 4:** The codes and subthemes pertaining to the theme of defects in the conceptualization of spirituality.

Theme	Subtheme	Code
Defects in the conceptualization of spirituality	Challenges related to the concept	God-orientation
		The different perspectives of different religions
		Disagreement about human existence
		No distinctions in meaning
		Different from religiosity
		Objectifying view
		Concerns about the narrow-mindedness
		Confusing with the topics of psychology
		Distortion of spiritual terminology

Defects in the conceptualization of spirituality	Necessity for spirituality conceptualization	Knowledge of humans
		Clear definition
		Distinction from ethics and psychology
		Consensus
		Definition of spiritual behaviors

Defects in the conceptualization of spirituality	Measurement of spirituality	Immeasurability
		Measuring its components
		Measuring its consequences
		Identifying with its manifestations

**Table 5:** The codes and subthemes pertaining to the theme of spirituality in practice.

Theme	Subtheme	Code
Spirituality in practice	Consequences of spirituality	Good feelings about life
		Satisfaction with life
		Surrendering to God's will
		The experience of love
		A balanced life
		The absence of distress
		Lasting peace of mind
		Avoiding ignorance
		Guarantee of true health
		Avoiding tensions
		Guaranteeing health in the hereafter
		Justifying the suffering

Spirituality in practice	Manifestations of spirituality	Respect
		Hopefulness
		Religious rituals
		Sacrifice
		Patience
		Faith
		Forgiveness
		Generosity
		Dedication
		Endurance of hardships
		Maintaining dignity
		Ethics
		Praying
		Healing
		Humanity
		Altruism

Spirituality in practice	Levels of spirituality	Being born with healthy spirituality
		The levels of adherence to religion
		Proper religious adherence
		Closeness to God (Allah)
